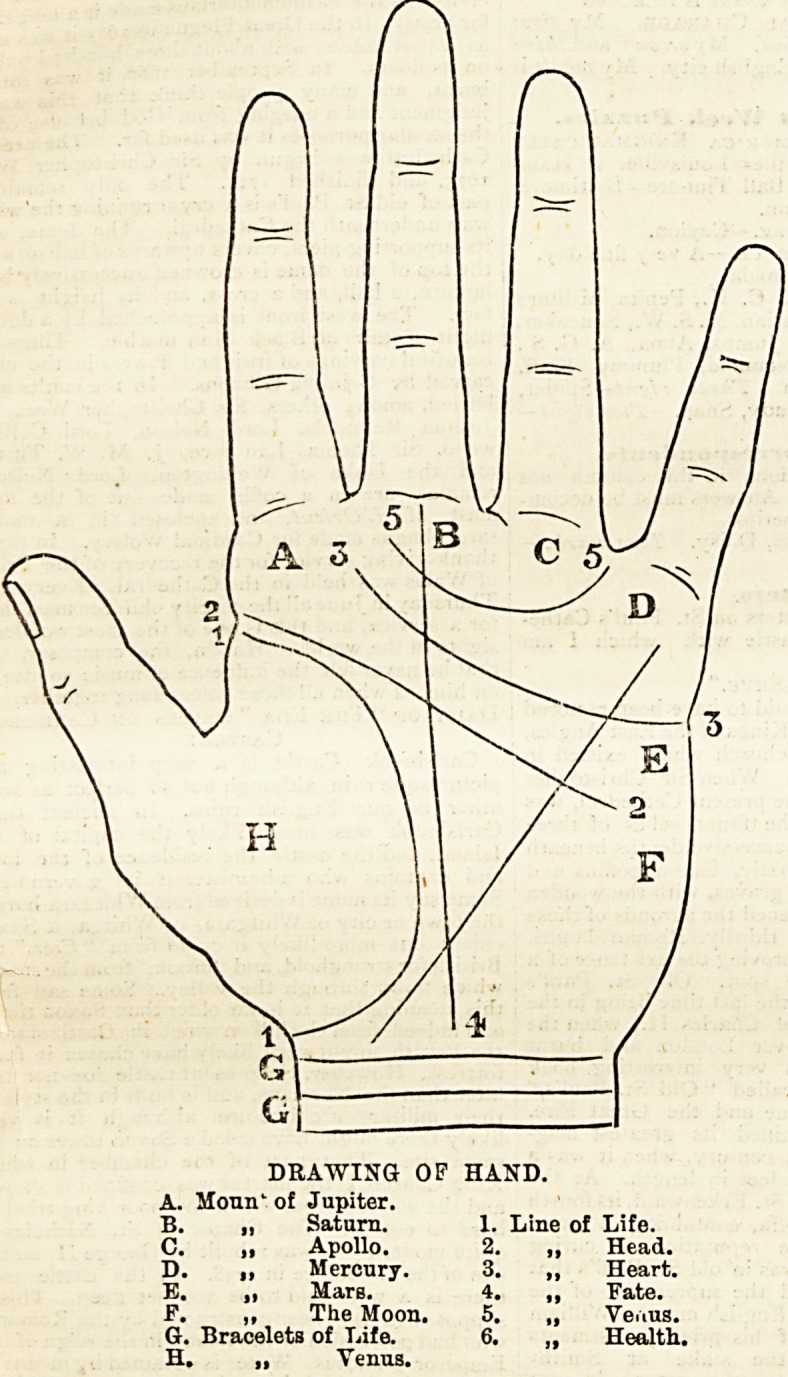# Palmistry Notes

**Published:** 1887-12-17

**Authors:** 


### AMUSEMENTS FOR CONVALESCENTS.

### PALMISTRY NOTES.

BY A LADY.

(*Continued from page 124.*)

DRAWING OF HAND.

| | |
|---|---|
| A. Mount of Jupiter. | |
| B. „ Saturn. | 1. Line of Life. |
| C. „ Apollo. | 2. „ Head. |
| D. „ Mercury. | 3. „ Heart. |
| E. „ Mars. | 4. „ Fate. |
| F. „ The Moon. | 5. „ Venus. |
| G. Bracelets of Life. | 6. „ Health. |
| H. „ Venus. | |

**Mounds of the Palm.**

WE now turn to the mounds of the palm to see what they can tell us, but first let me remind my readers that, although certain qualities are connected with the development of each mound, it entirely depends upon the shape of the fingers, the consistency of the hand, and the strength or weakness of the thumb as to how those qualities will be exercised and controlled. I have said elsewhere that a *soft hand indicates indolence and a hard one vigour of temperament.* A long first joint to thumb gives strength of *will*, and a long second phalange strength of *mind*, while it depends very much upon the shape of our fingers as to what form the attributes of the mount will take. For instance, with square or spatulate fingers our actions will be the result of reason and good sense, even if we are prone to regard things a little too much in their matter-of-fact every-day aspect; with pointed and conical fingers we shall possess more subtlety and imagination and instinctively idealize all things with which we come into contact. In reading the mounts, then, we must take the shape of the fingers and the other points I have named into consideration, as these are small matters to which it is very neceessary attention should be paid.

Beneath each finger a small swelling or cushion called a mound will be found which takes its name from one of the planets. At the base of the first finger lies the *Mount of Jupiter* (A). Well developed in a good hand, it gives us warmth of heart, liberality, reverence, and ambition for the highest good. With this mount high in our hands, we shall be self-reliant, daring, easy in bearing, with a certain dignity of demeanour, which will not detract from the social gifts characteristic of the mount. Open-handed and open-hearted, able to converse well, we shall delight in entertaining our friends, and at the same time be very susceptible of their admiration and good opinion. With no development of the mount, there will be an absence of the qualities named, and coldness and dulness will replace these lively characteristics; too full a development always shows a tendency to exaggeration, which will militate against the exercise of the better attributes of a mount; and these indications apply to any mount whose condition is one of deficiency or excess.

Lines are generally found on a mount, and according to their position and distinction, we judge of their significance. One clear, strong line on *Jupiter*, indicates great good fortune; a number of little cross-line obstacles, towards the attainment of our ambition; a cross foretells a happy marriage, and with a star in the vicinity, it will be one of distinction and wealth also. *The Mount of Saturn* (B). The characteristics of Saturn are in direct opposition to those of Jupiter. Here we find that caution, timidity, love of retirement, melancholy, and self-absorption, are the not very attractive qualities associated with the mount. If we belong to the Saturnian Type, in addition to the prominence of the mount in our hands, we should have long bony fingers (reason and philosophy) to aid us in the cultivation of those traits inseparable from the mount. Instead of being genial, active, self-reliant, and daring, qualities which shine in the foreground of life, we shall prefer a quiet, even existence, and not being particularly sociable, and somewhat fastidious, a little faddy even, sceptical also, and content with our own society, and, indifferent to to the impression we make on others, we shall strike out a line of life to suit our tastes and inclinations. Our self-sufficiency will make society unnecessary to us, and there is a strong probability that we shall end our days as old maids and bachelors. The charms of agriculture, gardening, botany, with little amateur dabbling in science, will have their attractions for us, and, if with Saturn, Mercury (E) is developed, it indicates a talent for medicine.

One line on the mount is a lucky omen, a number of little ladder-like lines extending to Jupiter foretells a gradual rise in life. A ray from the heart line (3) continuing to Saturn shows constant toil and anxiety, but if it end in a clear line on the mount, wealth and renown will be the result. A spot on the mount indicates a misfortune to be guarded against; if connected by a ray with the line of the heart, some disappointment or sorrow is the cause of it; if with the head line (2) injury or illness affecting the brain is to be feared.

We now come to *Apollo* (C) the mount of art and inspiration. With this clearly defined, and accompanied by a good line of Apollo cutting the mount love of and success in art, accompanied by wealth and good fortune will be ours. Combined with originality, ambition, and the intellectual gifts connected with the development of the mount, it will fit us for aspiring to distinction in more than one department of life. We shall spurn the idea of unmerited praise, but value the just appreciation of those who are competent to form an opinion of our powers. In religion our tendencies will be towards beauty and order in worship. An *excessive* development of the mount gives indolence, extravagance, jealousy, vanity, and inconstancy when combined with twisted fingers and a soft palm. A star on the mount indicates *without* the *line* of *Apollo*, riches without peace of mind; with the line, renown and celebrity as the result of talent and industry combined. Two lines signify unusual, but not always successful, talent. A spot on the mount indicates loss of character and position. Mercury (D) combined with Apollo gives force and strength to the artistic instincts, with eloquence, love of truth, and scientific research. The mounds of Venus (H) and Apollo developed kindliness and amiability.

(*To be continued.*)

## Figures and Tables

**Figure f1:**